# A prevalence study of Autism Spectrum Disorder in Russia

**DOI:** 10.3389/fpsyt.2026.1790306

**Published:** 2026-04-10

**Authors:** Oksana I. Talantseva, Raisa S. Romanova, Julia E. Kuznetsova, Viktoria A. Manasevich, Katerina V. Lind, Mariia A. Ivashchenko, Julia Benoit, Elena L. Grigorenko

**Affiliations:** 1 Center for Cognitive Sciences, Sirius University of Science and Technology, Sirius, Russia; 2 Department of Psychology, University of Haifa, Haifa, Israel; 3 Federal Resource Center for ASD, Moscow State University of Psychology and Education, Moscow, Russia; 4 Veltischev Research and Clinical Institute for Pediatrics and Pediatric Surgery of the Pirogov Russian National Research Medical University, Moscow, Russia; 5 Texas Institute for Measurement, Evaluation, and Statistics, University of Houston, Houston, TX, United States; 6 Department of Psychology, University of Houston, Houston, TX, United States; 7 Department of Molecular and Human Genetics, Baylor College of Medicine, Houston, TX, United States; 8 Department of Pediatrics, Baylor College of Medicine, Houston, TX, United States

**Keywords:** Autism Spectrum Disorders, epidemiology, prevalence, Russia, two-phase design

## Abstract

**Introduction:**

Autism Spectrum Disorder (ASD) is a neurodevelopmental condition marked by heterogeneity in presentation. In recent decades, ASD prevalence has risen globally, yet data from non-Western and middle-income countries, including Russia, remain scarce. Thus, Russian official statistics report a prevalence of 0.41 per 1,000 (0.041%), far below global estimates of 32.2 per 1,000 (3.2%), indicating underdiagnosis. This study provides the first population-based estimate of ASD prevalence among Russian elementary school children and examines barriers to diagnosis and treatment.

**Methods:**

The study followed a two-phase epidemiological approach and utilized well-known instruments (SCQ, ADOS-2, ADI-R) for ASD identification. The target population included all students in grades 1–3 (n = 34,847), stratified into mainstream, special education, and resource classes. The diagnostic phase had a 25.6% participation rate. ASD prevalence was estimated using Bayesian regression correcting for screening misclassification.

**Results:**

The estimated ASD prevalence was 22.2 per 1,000 children (95% CIs: 18.6–36.0), substantially exceeding what is reported based on the RF administrative data. ASD was more frequently identified in students from special education and resource classes than in general education settings. A Bayesian sensitivity analysis confirmed the robustness of prevalence estimates across plausible values for sensitivity and specificity.

**Discussion:**

These findings suggest that ASD prevalence in Russia is considerably underestimated via administrative data. Systemic barriers—including limited access to diagnostic services and stigma surrounding psychiatric labels—may hinder ASD identification. Addressing these challenges is essential for improving early detection and service provision in Russia and similar contexts.

## 1 Introduction

Autism Spectrum Disorder (ASD) is a lifelong neurodevelopmental condition characterized by social communication impairments and restricted and/or repetitive behaviors and interests (1, 2). The diversity of manifestations across these domains results in a striking heterogeneity in ASD presentation (3). This heterogeneity is further compounded by wide variability in intellectual and language functioning, intra-individual cognitive discrepancies (4, 5), and frequent comorbidity with other developmental and psychiatric conditions (6). Collectively, these factors place ASD among the most disabling developmental disorders, contributing to substantial economic burden and necessitating varied health service utilization (7).

Since the 1970s, ASD prevalence studies have been conducted in at least 37 countries—primarily high-income ones—while data from many low- and middle-income countries remain scarce (8). As a whole, these studies have demonstrated a substantial increase in ASD prevalence estimates worldwide since the late 20th century. Notably, data from the Autism and Developmental Disabilities Monitoring (ADDM) Network of the USA CDC highlight this trend, with prevalence rates among American 8-year-olds rising from 6.7 per 1,000 in 2000 (9) to 32.2 per 1,000 in 2022 (10), indicating a 380.6% increase.

Although the reasons for the reported upward trend remain under discussion, a substantial body of research highlights that reported prevalence estimates do not necessarily reflect true rates. Instead, these estimates are shaped by multiple factors, including the progression and refinement of diagnostic criteria; advancements in screening; diagnostic and policy practices; increased awareness and advocacy for ASD; potential environmental alterations, and the quality of research (11). Furthermore, prevalence estimates vary by case ascertainment methods and geographic regions, adding to the complexity of accurately measuring true ASD rates. Specifically, a recent meta-analysis (12) identified geographical region and socio-economic status as significant moderators of prevalence estimates. The highest estimates were reported for high-income countries, while the lowest were observed for low-income ones. Even within the same high-income country and within a single study encompassing different regions, these discrepancies persist. For instance, the most recent ADDM Network estimates ranged from 9.7/1,000 in Texas to 53.1/1,000 in California, despite the Network’s ongoing and increasingly successful efforts to reduce regional discrepancies through improved policies (10).

In Russia, psychiatric diagnoses are still based on ICD-10, under which ASD is considered an umbrella term for the following formal diagnostic codes: F84.0 (Childhood Autism), F84.1 (Atypical Autism), F84.4 (Overactive Disorder Associated with Mental Retardation and Stereotyped Movements), F84.5 (Asperger Syndrome), F84.8 (Other Pervasive Developmental Disorders), and F84.9 (Pervasive Developmental Disorder, Unspecified). To date, no targeted research has been conducted on the prevalence of ASD in Russia, with the sole exception of a study that assessed ASD risk using a domestic screening tool (13) in a sample of children aged 18 to 48 months across nine regions. This study estimated the risk of ASD at 1.8 per 1,000 (0.18%), which is substantially lower than risk estimates obtained using established screening instruments worldwide (14). The latest available statistics of the State Psychiatric Service in 2022 (15) have indicated a prevalence of ASD in Russia at 0.41 cases per 1,000, or at 0.041%. This includes 0.40 cases of childhood and atypical autism and 0.007 cases of Asperger’s syndrome per 1,000. Among individuals officially diagnosed with autism, 78.2% were reported to have a disability, with 94.7% of these being children under 17. Compared with 2018 (0.32 cases per 1,000, or 0.03%), the estimated prevalence of ASD has increased by 85.5%, likely reflecting improvements in diagnostic approaches and detection of ASD within the country. A more prominent increase from 2018 to 2020 was registered among children (aged 0–14 years) and adolescents (aged 15–17 years): from 0.11 to 0.17 cases and from 0.06 to 0.13 cases per 1,000, respectively (15). A retrospective study analyzing trends in governmental statistics on registered cases of ASD has also revealed a steady upward trend — critically delayed compared to worldwide estimates — observed in Russia since 2014 (16). Yet, despite recent improvements in detection and reporting, the reported (administrative) prevalence of ASD in Russia remains extremely low — approximately 29 times lower than the conservative global median rate (11). Thus, it is likely that a significant proportion of people with autism in Russia do not receive a diagnosis and, consequently, cannot access services. Consistent with other nationally representative samples, significant regional differences have also been observed, though these appear substantially uneven in their distribution. In 2021, estimates ranged from 0.17/10,000 in the Kaluga region to 17.77/10,000 cases in Yamalo-Nenets Autonomous Okrug (16). Given this disparity, autistic individuals and their families may find themselves in a challenging situation, often having to relocate in pursuit of an accurate diagnosis and appropriate services.

Here we report the first population-based prevalence study in Russia. We targeted the entire elementary school population in a large city, covering both a mainstream population sample and groups with a high risk of ASD. We also provide available administrative data reporting officially diagnosed ASD cases in the school-based population of the same city.

## 2 Materials and methods

### 2.1 Study site

The study was conducted in a large city that serves as the administrative center of one of Russia’s 89 federal subjects, located in the Central Federal District (one of eight federal districts in the country). As of January 1, 2023, this federal subject (oblast) had a population of 2,285,300, with the city itself accounting for 1,061,108 residents. To date, this city is considered one of Russia’s more progressive cities in terms of state-supported ASD-related services. Since 2013, the interdepartmental project *Autism: Routes of Assistance* has implemented early ASD risk screening, diagnostic services, and structured educational programs in the city. We selected this site as it ensures access to state-funded ASD care. In practice, children who received an ASD diagnosis in this study were referred to local providers for clinical confirmation and services.

### 2.2 Study population

The target population consisted of all 1^st^-, 2^nd^-, and 3^rd^-grade students attending mainstream or special education schools in the city during the 2023–2024 academic year (N = 34,847; 17,732 boys). It was divided into three separate strata, depending on the risk of having ASD. Different sampling strategies were employed for each stratum.

#### 2.2.1 Low-risk (LR) subpopulation

The LR target subpopulation (N = 34,392) comprised all children attending mainstream elementary schools (N = 118), except for schools that participated in the pilot study (N = 11). Given the large number of students in this subpopulation and the fact that a number (N = 18) of mainstream schools have resource classes for children with ASD, we used a mixed sampling strategy. First, all schools with resource classes were invited to participate in the study. Then, we randomly selected schools without resource classes based on the assumption that together, they would provide an adequate representation of all schools. The final sample size was determined based on a judgment of the optimal balance between the minimum sufficient sample size estimate, the research team’s limited resources, and results of the pilot study. Overall, 30 schools were selected (N = 8,565; 51.7% boys), covering 24.9% of the LR target subpopulation.

#### 2.2.2 Elevated-risk (ER) subpopulation

The ER subpopulation included all elementary students attending special education schools (N = 5), implementing programs for children with developmental disabilities (N = 292; 65.4% boys). Given the significant challenges in ASD diagnostics in Russia and the tendency of psychiatrists to assign only a single diagnosis without considering potential comorbidities (16), we assumed that this subpopulation might have an increased risk of ASD as well as a higher likelihood of misdiagnosis. Due to the relatively small size of this subpopulation, all relevant schools were invited to participate in the study. Thus, a convenience sampling design was applied.

#### 2.2.3 High-risk (HR) subpopulation

All children enrolled in resource classes (N = 112; 70.5% boys) were classified within the HR stratum. Since enrollment in resource classes requires both a medical and an educational diagnosis of ASD, we considered the risk of ASD in this subpopulation to be extremely high—approaching 100%. A convenience sampling design was also applied within the HR subpopulation: all schools were sought as eligible and invited to participate, and all children and their parents were recruited.

A detailed breakdown of the total number of schools in the study cite, including their distribution by type and participation status, is provided in **Supplementary Table 1**.

### 2.3 Case identification

**Figure 1:**
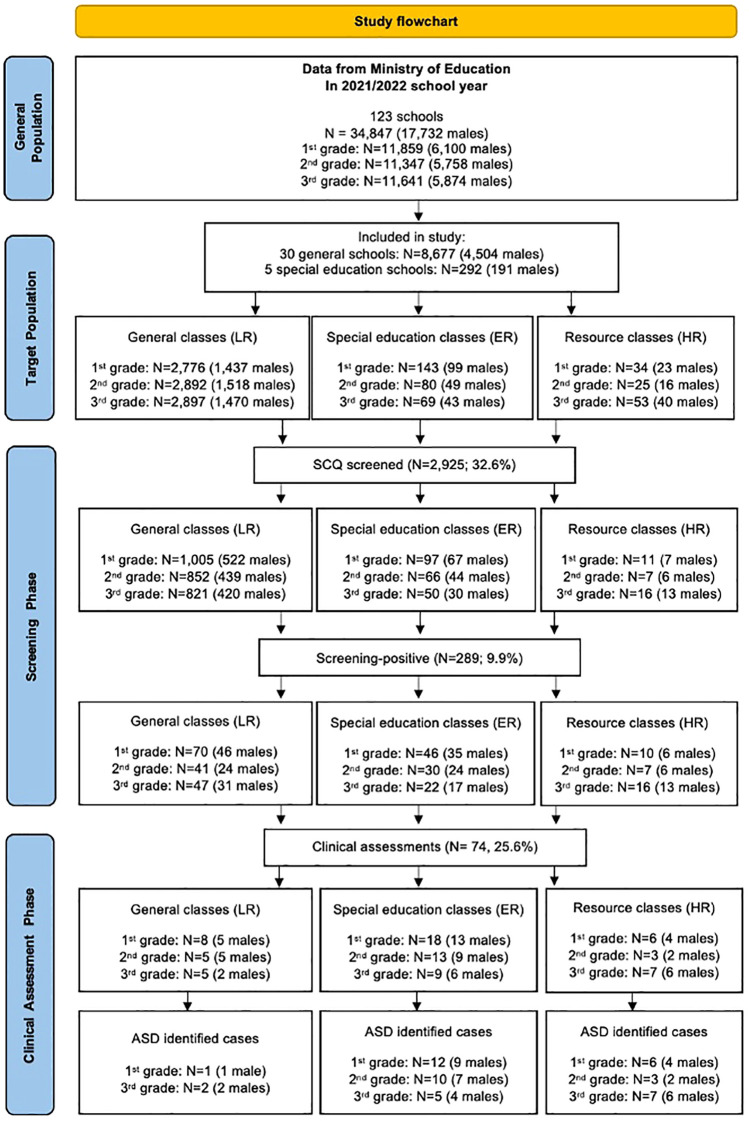
Flow-chart describing the case identification process across the 2 phases of the study.

To identify cases of ASD, we applied a two-phase design—screening and identification of children at risk for ASD, followed by clinical assessment and diagnostic confirmation of those who screened positive—regardless of strata (**Figure 1**).

#### 2.3.1 Screening phase

This phase was carried out from January 2023 to May 2024. To increase participation, pediatricians serving the selected schools were invited to act as interviewers during the first phase. Their on-site work was managed by a designated research team member, whose responsibilities included communicating with school principals, obtaining permission for school participation, and organizing the storage and delivery of completed packages of documents to the research team. Parent meetings were held separately at each school. Pediatricians attended these meetings, explained the aims of the study, distributed screening packages, encouraged participation, and answered any questions that arose. All participating pediatricians were trained by the research team and received monetary compensation for their support of the study.

Each envelope contained two identical informed consent forms (one copy was for the parents to keep), a consent form for personal data processing, and a screening questionnaire (Social-Communication Questionnaire, SCQ, Lifetime version) (17). Each form had an ID sticker; the SCQ forms did not include any identification information. Parents who declined to participate in the screening or did not sign the informed consent were considered non-respondents.

All these forms were completed at administrative meetings. The envelopes with the forms were returned to the research team for data entry, which was carried out with integrated quality control procedures. Randomly selected ten percent of the data were double-entered, and another ten percent of SCQ forms were checked for consistency against the printed copies.

Participants who scored ≥11 on the SCQ were classified as screening-positive and invited to the diagnostic confirmation phase. Although a cutoff score of 15 is commonly recommended to identify children at risk in clinical settings, the SCQ in the present study was used exclusively as a first-stage screening tool within a two-phase epidemiological design. In such designs, maximizing sensitivity at the screening stage is methodologically preferable in order to reduce potential false negatives, particularly when all screen-positive cases undergo comprehensive diagnostic assessment using gold-standard instruments (ADOS-2 and ADI-R). Previous studies have suggested that lower thresholds (e.g., 11–13) may be more appropriate in epidemiological contexts to improve sensitivity (18). Given the absence of large-scale psychometric validation studies establishing an empirically derived cutoff for the Russian-language version of the SCQ, and because diagnostic verification among screen-negative children was not feasible, we selected a threshold of ≥11 to prioritize sensitivity and minimize potential under-ascertainment at the screening stage.

Initially, we planned to sample both screening-positive and screening-negative groups with unequal probabilities during the Diagnostic Confirmation Phase, in order to estimate the proportion of ASD cases in each group and incorporate these estimates into the prevalence analysis, adjusted for non-response. However, constraints related to study duration, funding, and organizational challenges prevented us from following this protocol. As a result, only screening-positive cases were eligible for recruitment in the second phase.

#### 2.3.2 Diagnostic confirmation phase

This phase was carried out between July 2023 and November 2024. All screen-positive children were eligible for diagnostic confirmation. Families were approached for recruitment when a parent/guardian had provided permission to be contacted and valid contact information was available. Recruitment efforts included two text messages sent several days apart, followed by up to two phone calls if there was no response. Non-participation at this stage was treated as non-response rather than exclusion. Reasons for non-response included: (1) no phone number provided; (2) an incorrect or disconnected phone number; and (3) no response to phone calls or text messages after four documented contact attempts by the recruiter. Families who declined participation or could not be reached did not complete diagnostic evaluation, resulting in missing diagnostic outcomes.

Evaluation of the autism-related features was performed using The Autism Diagnostic Observation Schedule, Second Edition (ADOS-2) (19) and The Autism Diagnostic Interview - Revised (ADI-R) (20). Cognitive development was assessed via The Leiter International Performance Scale, Third Edition (Leiter-3) (21), adaptive functioning – through The Vineland Adaptive Behavior Scales, Second Edition (Vineland-II) (22). Multiple visits were required to complete this battery. During the first visit, the research team provided parents with detailed information about all planned procedures, and parents were asked to sign an informed consent to participate in the second phase of the study.

According to the original study protocol, a comprehensive diagnostic battery was planned to be used. However, in the pilot study, the number of refusals to participate increased with each additional assessment method. As a result, the ADOS-2 and SCQ-retest were made mandatory, while the remaining assessments were made voluntary.

All clinical assessments were conducted by the research team and administered at government and private centers that provide services for children with ASD. Upon parental request, the research team also provided written feedback, including descriptions of the child’s test scores and behaviors.

The final diagnostic decision was made in two steps. In the first step, the results of the assessment instruments were coded separately, and a preliminary diagnosis was made based on whether the child met the algorithm criteria of the diagnostic assessments. A diagnosis of ASD was given if the threshold score was met or exceeded, and symptoms were present across all relevant diagnostic domains. In the second step, the case was reviewed by considering the results of both assessments together. If there was a discrepancy between ADOS-2 and ADI-R outcomes, a consensus diagnosis was reached in conjunction with the clinical judgment of the research team, following DSM-5-TR diagnostic criteria.

Detailed information on all screening and diagnostic instruments used during both phases of the study is provided in the **Supplementary Appendix**.

### 2.4 Statistical analysis

To assess potential bias due to nonparticipation in the second phase, we first examined predictors of participation among screen-positive children. We hypothesized that families differ in their underlying willingness to pursue diagnostic evaluation, and that this latent trait may be partially explained by observable characteristics. Using logistic regression, we found that SCQ scores were significantly associated with participation (*p* <.05), whereas sex and age were not (*p* = .83 and *p* = .76, respectively). This indicates that participation was not random but associated with symptom severity. This finding justified the inclusion of the SCQ score as a covariate in subsequent models.

To estimate ASD prevalence while accounting for missing diagnostic data and misclassification, we implemented a Bayesian logistic regression model in which ASD status was treated as a latent binary variable. Observed diagnostic outcomes were modeled as error-prone measurements of this latent status, conditional on known values of screening sensitivity (Se) and specificity (Sp). The probability of a positive diagnosis was thus a function of both the unobserved true ASD status and the psychometric properties of the screening tool, following established frameworks for probabilistic bias analysis (23) and Bayesian modelling of misclassified outcomes (24). The individual probability of true ASD was modeled as a function of SCQ score.

Importantly, because SCQ score was associated with participation and was simultaneously entered in the latent outcome model, the estimation procedure implicitly adjusted for differential participation. Posterior ASD probabilities were then estimated for all eligible screen-positive children—including those who did not attend the diagnostic phase—based on their SCQ scores and the estimated model parameters. Thus, nonparticipants were not excluded from inference but were incorporated through probabilistic imputation within the hierarchical model.

In the primary analysis, Se and Sp were fixed at 0.85 and 0.75, respectively, based on published validation studies (19). The model was implemented in R using the *rjags* package (25) with three MCMC chains, 5,000 burn-in, and 10,000 post-burn-in iterations. Posterior ASD probabilities were aggregated within each stratum (LR, ER, HR) and combined using known population sizes to produce post-stratified prevalence estimates. Posterior distributions were summarized using median and 95% Bayesian credible intervals (CrIs).

To assess robustness, we conducted deterministic sensitivity analysis across 16 plausible combinations of Se (0.80–0.95) and Sp (0.65–0.80), based on values reported in previous SCQ validation studies (16, 17). Additionally, a probabilistic sensitivity analysis treated Se and Sp as uncertain parameters with informative Beta priors (Se ~ Beta(85,15); Sp ~ Beta(60,40)). We also explored the influence of assumptions in the HR stratum by constraining its posterior prevalence to 100%, 95%, 90%, or 85%. These analyses allowed us to evaluate the stability of prevalence estimates under varying assumptions about screening performance and model structure. Additionally, a probabilistic sensitivity analysis treated Se and Sp as uncertain parameters with informative Beta priors (Se ~ Beta(85,15); Sp ~ Beta(60,40)). Under the standard Beta(a,b) parameterization, these correspond to prior means of 0.85 and 0.60, respectively (E[θ]=a/(a+b)). We selected a more conservative prior for specificity to reflect greater uncertainty under the lowered SCQ cutoff (≥11) and the absence of large-scale validation of the Russian SCQ. Full details of this analysis, as well as complete model specifications, are provided in the **Supplementary Appendix**.

To assess model convergence and numerical stability, we evaluated standard MCMC diagnostics. Convergence was examined using Gelman–Rubin potential scale reduction factors ( $\hat{\text{R}}$), and effective sample sizes (ESS) were computed for key regression parameters. Trace plots and posterior density plots were visually inspected. Overall, diagnostics indicated acceptable convergence of the primary regression parameters and adequate numerical stability of the posterior estimates. Detailed diagnostic results are provided in the **Supplementary Appendix**.

### 2.5 Administrative prevalence

In addition to the main study, we also requested statistical data on diagnoses compatible with ASD (ICD-10 codes F84.0, F84.1, F84.4, F84.5, F84.8, and F84.9) that were registered in the city’s educational system among elementary school students from 2020 to 2024. Prevalence estimates were calculated as simple proportions, dividing the number of cases by the size of the target population.

## 3 Results

Thirty-five school (including five special education schools and ten with resource classes) participated, yielding a total target population of 8,976 children. The distribution of SCQ scores across strata is summarized in **Supplementary Table 2**. Screening-positive proportions differed across strata, with the highest rates observed in the HR group. The overall response rate was 32.6% in the screening phase and 25·6% in the diagnostic phase, with lower participation in mainstream settings (see **Supplementary Table 3** for stage-specific participation by stratum). The flow of participants through the study is described in **Figure 1**.

The pooled prevalence adjusted for participation bias was 15.9 per 1,000. Bayesian correction for misclassification yielded a post-stratified prevalence of 22.2 per 1,000 (95% CrI: 18.6–36.0), with adjusted sex-specific rates of 24.7 per 1,000 in males and 17.7 per 1,000 in females. The empirical sex ratio of 3.2:1 was reduced to 1.4:1 after adjustment. Detailed prevalence results are provided in **Table 1**.

**Table 1:** ASD prevalence estimates by sex and grade.

Grade	Population		N cases		Sex ratio	Prognosed cases		Prevalence (95% CrIs) per 1,000		
	M	F	M	F		M	F	M	F	Overall
1^st^ Grade	596	517	14	5	2·8	14	9	25·6 (20·5; 45·4)	17·5 (15·5; 30·6)	22·4 (18·6; 38·5)
2^nd^ Grade	489	436	9	4	2·3	10	10	22·5 (18·9; 32·7)	24·3 (19·9; 33·5)	23·3 (19·9; 32·9)
3^rd^ Grade	462	425	12	2	6·0	12	4	26·0 (21·6; 45·2)	13·9 (9·7; 28·0)	20·7 (16·7; 36·2)
Overall	1547	1378	35	11	3·2	36	22	24.7 (20·3; 40·8)	17·7 (14·5; 29·5)	22·2 (18·6; 36·0)

ASD, autism spectrum disorder; M, males; F, females; CrIs, Credible Intervals.

**Figure 2:**
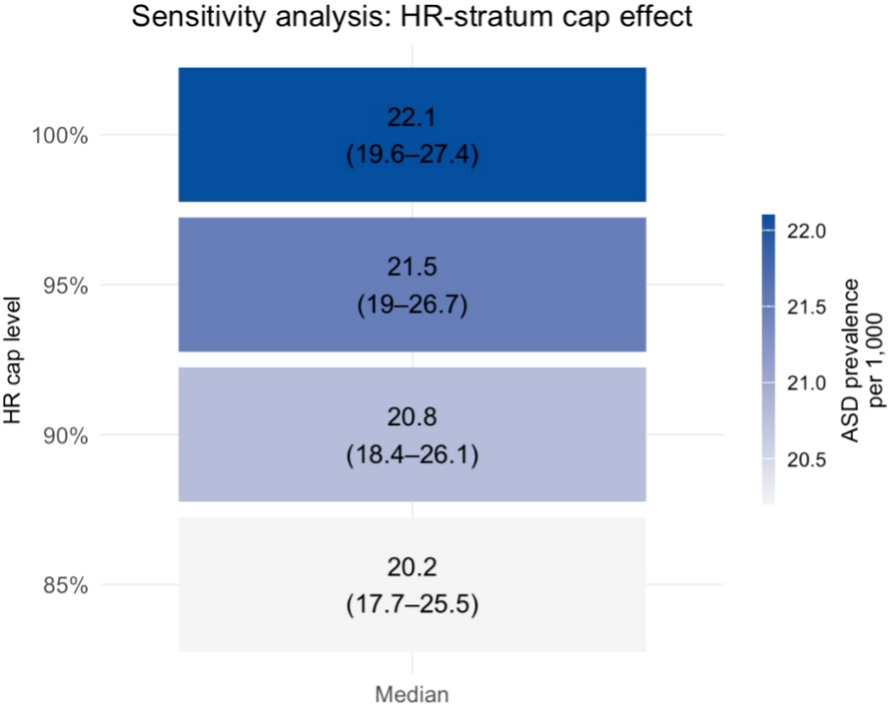
Post-stratified ASD prevalence estimates under varying screening sensitivity (Se) and specificity (Sp). Each cell shows the median ASD prevalence (%) obtained from deterministic sensitivity analysis using a Bayesian logistic regression model with corresponding 95% credible intervals shown in parentheses.

**Figure 3:**
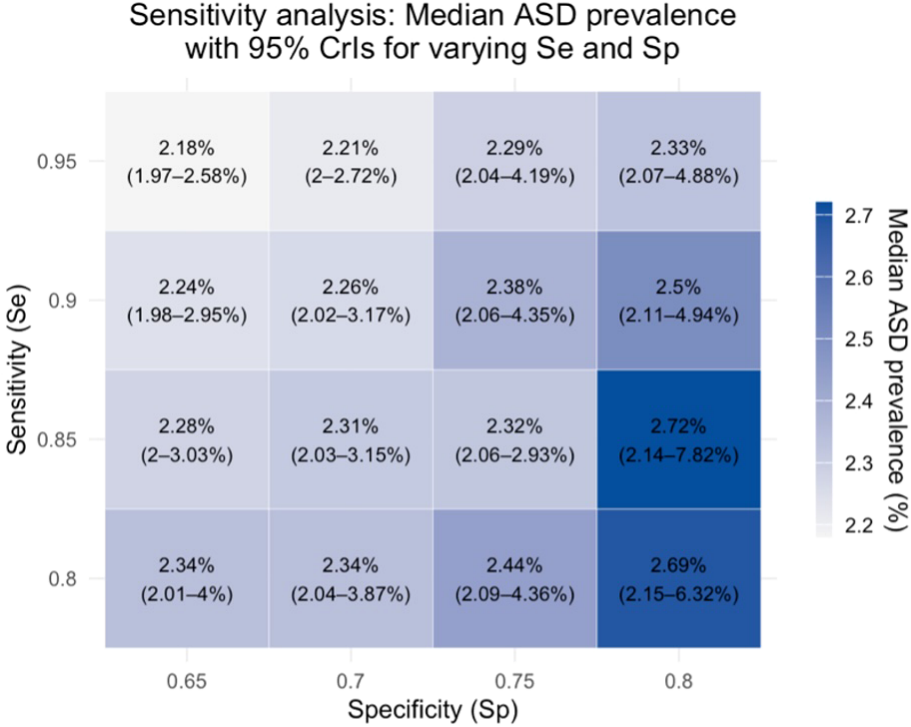
Sensitivity analysis of post-stratified ASD prevalence under varying upper bounds for the HR stratum. Each row represents a model scenario in which the predicted prevalence in the high-risk (HR) stratum was artificially capped at a maximum value (100%, 95%, 90%, or 85%). Cells show the median ASD prevalence per 1000, post-stratified across HR, IR, and LR strata. Color intensity reflects the magnitude of prevalence. The results show minimal variation (20.4–20.6 per 1000), supporting the robustness of the overall estimate.

Sensitivity analyses confirmed robustness of prevalence estimates across a range of plausible screening sensitivity and specificity values, as well as under conservative assumptions in the HR stratum (see **Figures 2**, **3**; **Supplementary Appendix** for full details).

Among participants diagnosed with ASD, intellectual functioning was assessed in 20 children. In the LR stratum, the children demonstrated average IQ (M = 101.5, SD = 0.7). In the ER stratum, 30% had average or above-average IQ, 30% were in the borderline range, and 40% met criteria for mild intellectual disability (M = 76.4, SD = 16.5). In the HR strata, one child (12.5%) had an average IQ, 50% were in the borderline range, and 37.5% had mild intellectual disability (M = 75.6, SD = 9.9).

Adaptive functioning was evaluated in 24 participants using the Vineland-II. In the LR group, one child had age-appropriate adaptive behavior, while the other showed mild deficits. In the ER group, one child (7.7%) had adequate functioning, two (15.4%) were borderline, and the majority (76.9%) demonstrated impairments, including one case with moderate deficits. In the HR group, one participant showed borderline functioning, and the remaining eight of nine children (88.9%) had impaired adaptive behavior, ranging from mild to moderate severity.

The prevalence of ASD derived from administrative data obtained from educational sources for the 2023–2024 academic year, was 4.1 cases per 1,000. Similar estimates (**Table 2**) were also observed for the two preceding academic years (4.1/1,000 and 4.0/1,000 in 2021–2022 and 2022-2023, respectively). In 2020-2021, the prevalence was slightly higher—5.0 cases per 1,000.

**Table 2:** Administrative ASD prevalence.

Year	2020-2021	2021-2022	2022-2023	2023-2024
ASD	194	166	164	174
Total	38 748	40 313	41 327	42 200
Prevalence per 1,000	5·0	4·1	4·0	4·1
95% CI	4·3-5·7	3·5-4·7	3·4-4·6	3·5-4·7

ASD, autism spectrum disorder.

## 4 Discussion

To our knowledge, this is the first population-based prevalence study of ASD in Russia. Based on direct clinical assessment, the estimated prevalence of ASD among elementary school students was 2.2%. This estimate exceeds the global consensus rate of 1% accepted by the World Health Organization (26) and is higher than summary estimates reported in recent meta-analyses (11, 12). At the same time, our findings are consistent with more recent international studies, many of which report prevalence rates approaching or exceeding 2%. For example, a recently published study reported an ASD prevalence in Cyprus at 1.8% (27). The result is comparable with the estimate reported in a study with a similar analysis approach to access the prevalence of ASD in South Korea (28). In comparison, our estimates fall below those from the U.S.-based ADDM Network, which reported prevalence rates of 2.8% and 3.2% in 2020 and 2022, respectively (10, 29). This discrepancy may reflect differences in methodology, as ADDM studies have been shown to yield higher estimates relative to population-based designs that rely on direct clinical evaluation (12).

The empirically observed male-to-female ratio in ASD prevalence was 3.2:1, aligning with recent literature that challenges the long-standing ratio of 4.0–4.5:1. Our findings support the revised estimate of approximately 3.0:1, as reported by Loomes et al. (30) and other recent studies accounting for diagnostic biases that disproportionately affect girls (11, 31). Notably, when adjusting for multiple potential biases using Bayesian hierarchical modeling—including screening misclassification, non-response, and sample structure—the male-to-female ratio in prevalence was further reduced to 1.4:1. This marked reduction should be interpreted with caution. The adjusted ratio does not necessarily imply that the true population sex ratio in ASD approaches 1.4:1, as such a low ratio has not been consistently reported in the broader international literature. Rather, the shift reflects the impact of model-based correction for differential participation and screening characteristics. Because SCQ scores were associated with participation in the diagnostic phase—and because symptom expression and recognition may differ by sex—probabilistic adjustment redistributed latent ASD probability among nonparticipants. As a result, part of the observed male predominance in the crude estimate may reflect ascertainment and participation effects rather than purely biological differences. Therefore, the adjusted ratio should be viewed as an estimate conditional on the modeling framework rather than as definitive evidence of a substantially lower underlying epidemiological sex ratio. This interpretation is further supported by recent large-scale population-based evidence demonstrating that the male-to-female ratio in ASD diagnosis is not stable across the lifespan but decreases with age and across more recent birth cohorts, approaching near parity in early adulthood (32), suggesting delayed or missed diagnosis in females. However, further studies incorporating full diagnostic verification across screening strata would be required to determine the true sex distribution in the population.

The prevalence raw from administrative data in the Russian educational system was substantially lower, ranging from 0.4% to 0.5% between 2020 and 2024. On the one hand, this discrepancy provides strong evidence of underdiagnosis in the school-age population and highlights the need to improve diagnostic services and awareness of ASD in Russia. On the other hand, such discrepancies between administrative and population-based estimates are common across countries (33), especially when administrative data rely on passive detection or incomplete records.

The current study has several limitations. The most notable is the low participation rate in the diagnostic confirmation phase (25.6%), which may have introduced selection bias. Despite substantial organizational support from regional health and education authorities, many parents were reluctant to engage in a study focused on psychiatric diagnosis. Stigma surrounding mental health conditions, reluctance to associate one’s child with a diagnostic label, and limited familiarity with research participation likely influenced consent decisions. Participation patterns differed across strata: families of children in specialized schools were more likely to respond, whereas families of children in resource classes—where a formal ASD diagnosis was often already established—were less motivated to attend further evaluation. To mitigate potential bias due to non-response, prevalence estimation incorporated covariate-adjusted modeling of participation as a function of SCQ scores, as well as post-stratification to known population structures. Nevertheless, residual selection effects cannot be fully ruled out, and prevalence estimates should be interpreted with this limitation in mind.

A second limitation concerns the two-phase design, in which only children scoring above the SCQ screening threshold were eligible for diagnostic evaluation. As a result, false-negative rates among screen-negative children could not be empirically estimated and were addressed through modeling assumptions regarding screening sensitivity and specificity. Although the Russian-language SCQ has not undergone large-scale psychometric validation, and a lower cutoff (≥11) was used to prioritize sensitivity, our findings remained robust across a wide range of plausible screening performance parameters. Deterministic sensitivity analyses varying sensitivity (0.80–0.95) and specificity (0.65–0.80) produced minimal fluctuation in post-stratified prevalence estimates. In addition, probabilistic sensitivity analysis utilizing informative Beta priors yielded highly comparable posterior prevalence distributions.

An additional important limitation is that diagnostic verification was not conducted among children who screened negative on the SCQ. Although the original study protocol planned to sample a subset of screen-negative participants, this component could not be implemented due to logistical and organizational constraints. As a result, the study does not allow empirical estimation of false-negative rates within the Russian context, and prevalence estimates therefore rely on assumptions about the screening instrument’s performance.

Because prevalence was estimated using a Bayesian misclassification framework, the results should be interpreted as conditional on the specified screening parameters and model structure. In the absence of empirical verification among screen-negative children, sensitivity and specificity were informed by prior evidence and incorporated either as fixed values or as informative prior distributions. Importantly, the overall magnitude of the prevalence estimate remained stable across a broad range of plausible screening assumptions, suggesting that the findings are not driven by a single parameter choice, even though the exact point estimate remains model-dependent. As with all analyses correcting for imperfect ascertainment, the reported prevalence should be viewed as the best estimate under clearly defined assumptions rather than as an assumption-free empirical quantity. If screening sensitivity was substantially lower than assumed, the true prevalence could be underestimated; conversely, overestimation of sensitivity could inflate model-based correction. To address this, we explicitly incorporated screening misclassification into the Bayesian model and conducted extensive deterministic and probabilistic sensitivity analyses across a broad range of plausible values.

Despite these limitations, we believe that our results—interpreted with appropriate caution—offer important insights into the burden of ASD in Russia. They can inform health-system planning and resource allocation, highlight regional disparities in diagnostic coverage, and guide future epidemiological efforts to identify social or environmental risk factors. As the first study of its kind in the Russian context, these findings help fill a geographic gap in global ASD prevalence research and contribute essential data to future meta-analyses and global surveillance efforts.

## Data Availability

The datasets used and analyzed during the current study available from the corresponding author on reasonable request. All R scripts used for Bayesian prevalence estimation, including model specification, post-stratification, and sensitivity analyses (deterministic and probabilistic), are available at: https://github.com/OksanaTalantseva/prevalence-asd-russia/tree/main. Requests to access the datasets should be directed to Elena.Grigorenko@times.uh.edu.
