# Supplementary material for: A prevalence study of Autism Spectrum Disorder in Russia
Source: Front Psychiatry. 2026 Apr 10;17:1790306. doi: 10.3389/fpsyt.2026.1790306 (PMC13106449; doi:10.3389/fpsyt.2026.1790306)
Supplement: Supplementary file 1 [file Supplementaryfile1.docx]

**Supplementary Appendix**

**Methods**

**Study Site**

According to the Educational Law in Russia, school enrollment is compulsory for all children. Children are admitted to primary school only after the age of six and a half in the absence of any disabilities or health problems and no later than the age of eight. If developmental concerns are present, the child is referred to an interdisciplinary team of specialists (psychological-medical-educational commission) for a comprehensive medical and diagnostic assessment and following decision on a type of recommended educational program. Following this, the parents of a child are permitted to choose a school and a program type. Hence, a child with confirmed special educational needs may also attend a mainstream educational class with needed accommodations and individual educational plan. However, many parents of children with developmental disabilities choose special educational schools. In Russia, they are divided into diagnostic groups (schools for children with hearing or visual impairments, and developmental delays, among others). The most appropriate form of education for children with ASD in Russia is the so-called “resource class” (commonly referred to as a «resource room»). In this case, the child is assigned to a mainstream educational class, but he or she attends part of the compulsory lessons in a specialized class where, in addition to learning disciplines, a child is also taught behavioral and socialization skills. Assigning a child to a resource class requires a medically confirmed diagnosis, as well as a decision of the special education commission, recommending this type of program (i.e. educational diagnosis). In the 2022–2023 academic year, 0.25% of students (326 individuals) were enrolled in adapted programs with both a medical and educational diagnosis of ASD.

**Sample Size Estimation**

To estimate the prevalence of a condition expected to affect approximately 1% of the population with adequate statistical precision, we calculated the required sample size based on the standard formula for estimating a single proportion with a specified level of confidence and absolute precision (1,2):

(1)

$$n=\frac{Z^{2}\cdot P\left( 1-P \right)}{d^{2}}$$

Where n is the required sample size, P is the expected prevalence (0.01), d is the desired absolute precision, and Z is the standard normal deviate corresponding to a 95% confidence level.

We considered two levels of precision, reflecting realistic operational goals and commonly recommended thresholds for low-prevalence studies (2,3):

- **±0.2% precision** (d=0.002);
- **±0.3% precision** (d=0.003).

For **±0.2% precision**, the required sample size was calculated as 9,508 participants. At an assumed prevalence of 1%, this would yield approximately 95 positive cases, with a resulting 95% confidence interval of **0.81% to 1.22%** based on the Clopper-Pearson exact method for binomial proportions.

For **±0.3% precision**, the required sample size was reduced to 4,226 participants. This sample would yield around 42 positive cases and provide an estimated 95% confidence interval of **0.72% to 1.34%.**

Thus, a sample size of approximately 9,000 participants was considered sufficient to ensure adequate statistical power to estimate a 1% prevalence with precision ranging from ±0.2% to ±0.3% at a 95% confidence level.

**Table 1 School Type, Participation, and Risk Stratum Allocation**

| Type of school | Risk Stratum | Total Schools | School Invited | Schools Participated | % of Total Schools |
| --- | --- | --- | --- | --- | --- |
| Mainstream public schools without resource classes | LR | 100 | 18 | 18 | 18% |
| Special education schools | EL | 5 | 5 | 5 | 100% |
| Mainstream public schools with resource classes | LR+HR | 18 | 18 | 12 | 66.7% |

*LR = low risk; EL = elevated risk; HR = high risk*

**Assessments**

The study used only Russian-language adapted versions of the following methods:

*The Social Communication Questionnaire (SCQ)*(4) was used as the screening tool for ASD. The SCQ is a 40-item questionnaire that assesses a range of behaviors associated with ASD, both currently and at age 4–5 years, across three domains: social interaction, language and communication, and repetitive/stereotypical behavior. Each item has two response options: ‘yes’ (the behavior is present) and ‘no’ (the behavior is absent). In our study, we used the ‘Lifetime Form’. The questionnaire takes up to 10 minutes to complete. The possible score ranges from 0 to 39 for verbal children and from 0 to 33 for nonverbal children. Although the authors recommend a cutoff score of 15 to define the at-risk group, we used a cutoff of 11 points. This decision was based on previous studies showing that a score between 11 and 13 is more optimal for epidemiological research, as it reduces the number of false negatives (5). This choice was also influenced by the fact that the Russian-language version of the SCQ has not yet been validated on a sufficiently large sample, suggesting that its cutoff score may differ from that of the original version.

*The Autism Diagnostic Observation Schedule, Second Edition (ADOS-2)* (6) is a comprehensive, researcher-administered procedure that places the child in realistic social situations designed to elicit behaviors specific to ASD. Although the protocol follows a standardized structure, the situations themselves are unstructured and do not dictate how the child should behave. Behaviors observed during the assessment are coded across the following domains: communication, social interaction, play and imagination, stereotypical or repetitive behaviors, and restricted interests. The instrument consists of five modules tailored to children of different ages and language development levels. In our study, we used Modules 1-3, depending on the child’s mental age. The ADOS-2 diagnostic algorithm is based on the criteria of the Diagnostic and Statistical Manual of Mental Disorders (DSM-V) and allows the determination of the severity of ASD symptoms.

*The Autism Diagnostic Interview - Revised (ADI-R)*(7) is a diagnostic tool consisting of a detailed, semi-structured clinical interview covering most aspects of development and behavior relevant to ASD. It includes questions designed to assess three main areas: the quality of social contact, communication and speech, and repetitive, restricted, or stereotyped interests and behavior patterns. The assessment is based on an algorithm aligned with the diagnostic criteria for autism in the DSM-IV. The ADI-R is conducted with a parent or legal representative of the child. In our study, trained assessors administered the interview remotely via video-calls.

*Leiter International Performance Scale, Third Edition (Leiter-3)* (8) is a comprehensive test of nonverbal intelligence and cognitive functions for children and adults aged three years and older. During the Leiter-3 assessment, all tasks are presented without the use of spoken language. The Leiter-3 includes two batteries of subtests. The cognitive subtest battery consists of five subtests of nonverbal intellectual abilities related to the processing of visual information and logical thinking. Four of these five subtests are necessary to determine the non-verbal intelligence quotient (IQ). The attention and memory subtest battery also includes five subtests: two for assessing non-verbal attention, two for memory, and one evaluating Stroop cognitive interference.

*The Vineland Adaptive Behavior Scales, Second Edition (Vineland-II)*(9) is a semi-structured interview in which the assessor asks questions answered by parents or individuals from the immediate environment of the person being evaluated. This interview assesses the level of adaptive functioning by evaluating behavior across four main domains: communication, daily living skills, socialization, and motor skills. The instrument also includes a scale for assessing maladaptive behavior.

**Statistical analysis**

We estimated the true prevalence of ASD using a Bayesian hierarchical logistic regression model implemented in RJAGS. The model accounted for three population strata — HR, ER, and LR — and incorporated individual-level SCQ screening scores as a covariate. Diagnostic outcomes were modeled as a latent (unobserved) binary variable for all eligible children, including those who did not participate in the Second-Phase diagnostic evaluation.

Observed diagnoses were treated as potentially misclassified measurements of the latent ASD status based on the known performance of the SCQ instrument. Specifically, the model incorporated screening sensitivity (Se = 0.85) and specificity (Sp = 0.75). The likelihood of an observed diagnosis *y_i_* was defined as:

| $y_{i} \sim Bernoulli\left( z_{i} \cdot Se + \left( 1 - z_{i} \right) \cdot\left( 1 - Sp \right) \right)$ | (1) |
| --- | --- |

Where *z_i_* is the true (latent) ASD status of an individual *i*. The individual-level diagnosis probability was modeled as:

| $logit\left( p_{i} \right)=\alpha+\beta\cdot{SCQ}_{i}$ | (3) |
| --- | --- |

Where *p_i_* is individual-level probability of true ASD for subject *i; α* is the intercept*, β* is the regression coefficient, *SCQ_i_* is the subject’s score on the SCQ.

The posterior distribution of individual ASD status (*z_i_*) was estimated via Markov Chain Monte Carlo sampling using 3 chains with 10,000 iterations each. This yielded full posterior distributions for each parameter, including individual diagnosis probabilities and overall ASD prevalence.

In addition to estimating the unweighted overall prevalence, we derived a post-stratified ASD prevalence estimate that reflected the true population structure across strata. Stratum-specific posterior prevalence distributions were first computed based on the imputed *z_i_* values within each group (HR, ER, LR). Then, using known target population sizes—HR (N = 112), ER (N = 292), LR (N = 8,684)—we calculated the weighted average prevalence at each iteration as:

| $\hat{P_{\text{total}}^{\left( j \right)}}=\frac{\sum_{g} N_{g}\cdot\hat{p_{g}^{\left( j \right)}}}{\sum_{g} N_{g}}$ | (4) |
| --- | --- |

Where $p_{g}^{\left( j \right)}$denotes the posterior prevalence in stratum *g* at iteration *j* and *N* is a stratum population size.

The posterior distributions of the parameters of interest, including overall and stratum-specific ASD prevalence, were summarized using their median and 95% Bayesian credible intervals (CrIs). Specifically, for each posterior distribution, we reported the 2.5th, 50th (median), and 97.5th percentiles as point and interval estimates. This non-parametric interval reflects the range within which the true parameter value falls with 95% probability, given the model and data.

To assess the robustness of our prevalence estimates to potential misclassification of ASD due to imperfect screening performance, we conducted the deterministic sensitivity analysis varying the sensitivity (Se) and specificity (Sp) of the SCQ. Empirically supported values were selected based on prior validation studies of the SCQ in both clinical and community samples. The analysis examined all 16 combinations of Se ∈ {0.80, 0.85, 0.90, 0.95} and Sp ∈ {0.65, 0.70, 0.75, 0.80}, covering the plausible range reported in the literature. For each combination, the Bayesian logistic regression model (with covariate-adjusted misclassification correction) was re-estimated. The resulting posterior median and 95% credible intervals (CrIs) of the post-stratified ASD prevalence were recorded. Following established recommendations, results were considered robust if the median prevalence estimates remained within a narrow absolute range (≤1% point) and the direction of conclusions was preserved across plausible parameter values. In addition, we implemented a probabilistic sensitivity analysis in which Se and Sp were treated as uncertain parameters and modeled using informative Beta priors (Se ~ Beta(85,15), Sp ~ Beta(60,40)). Under the standard Beta(a,b) parameterization (with mean a/(a+b)), these priors correspond to prior means of 0.85 for sensitivity and 0.60 for specificity. The choice of these distributions reflects empirically plausible screening performance values reported in the literature, while allowing for uncertainty, and incorporates a more conservative assumption regarding specificity given the lowered SCQ cut-off (≥11) and the absence of large-scale psychometric validation of the Russian-language version. These priors were incorporated into the Bayesian model to propagate misclassification uncertainty directly into prevalence estimation. Posterior distributions of Se, Sp, and ASD prevalence were derived from 10,000 Markov Chain Monte Carlo draws.

To further examine the potential influence of high-confidence model predictions in the HR stratum, we conducted a sensitivity analysis in which the posterior prevalence estimates for HR were constrained by imposing artificial upper bounds. Specifically, we truncated the iteration-level posterior values of HR prevalence to not exceed 100%, 95%, 90%, or 85%, while maintaining full model-based estimation in all strata.

This approach allowed us to test the extent to which strong model certainty in the HR group affected the overall post-stratified prevalence estimate, under increasingly conservative assumptions.

Model convergence was assessed using standard Markov chain Monte Carlo (MCMC) diagnostics. Four parallel chains with dispersed initial values were run with adaptation, burn-in, and post–burn-in sampling as described in the main Methods. Convergence was evaluated using the Gelman–Rubin potential scale reduction factor (R̂) (Gelman & Rubin, 1992) (10). Values of **R̂ < 1.10** were considered acceptable, and **R̂ ≤ 1.05** indicative of good convergence.  Monte Carlo sampling efficiency was evaluated using the effective sample size (ESS). For primary model parameters (regression coefficients and prevalence), **ESS > 400** was considered sufficient for stable posterior estimation, and **ESS > 1000** indicative of high precision (Vehtari et al, 2021) (11).

Posterior predictive checks were conducted by simulating replicated datasets from the posterior distribution and comparing observed and predicted proportions, following standard Bayesian model-checking procedures (Gelman et al., 2013) (12).

***Software and Computational Environment***

All statistical analyses were conducted using R version 4.3.2 (10). The following R packages were used: (1) *dply (13)* for data manipulation and filtering; (2) *stats (14)*for logistic regression modeling; (3) *boot (15)* for non-parametric bootstrap resampling and confidence interval estimation; (4*) rjags* (16) for Bayesian modeling via Markov chain Monte Carlo using JAGS; (5) *coda (17)* for convergence diagnostics and posterior summary statistics; and (6) *ggplot2* (18) for data visualization.

**Results**

***Screening Phase***

A total number of 2,964 packages of documents were returned by parents. After quality control, 39 packages were excluded due to lack of signed informed consent (N = 16), non-target grade (N = 14), non-target school (N = 4), non-identified school (N = 2), non-identified school program (N = 2), and refilling (N = 1). Thus, the total sample was 2,925 participants (1,548 males, 53%), and the mean age of the sample was 8.2 years old (SD = 1.0). The overall response rate for participation in the screening phase was 31.3%. Also, the response rate varied in different strata (31.2, 72.9, and 30.4% in LR, ER, and HR correspondingly). Reasons for refusal to participate were not recorded in this phase.

The mean total SCQ score was 5.4 (SD = 4.8). The population by strata, grade and mean SCQ results cohort distribution of the screened children are summarized in Table 2. As expected, the significant effect of the strata on screening results was revealed (*F(*2, 2,922) = 2,304, *p* < .001) with the lowest percentage of screening positive cases in the LR (7.0%), a notably higher percentage in the ER (46.0%) and the highest in the HR (96.2%) stratum.

**Table 2: Distribution of SCQ Scores and Screening Outcomes Across Strata and Grades**

|  | **N** | **Age, Mean (SD)** | **SCQ, Mean (SD)** | **SCQ ≥11, N (%)** | **SCQ ≥15, N (%)** |
| --- | --- | --- | --- | --- | --- |
| **All individuals** | 2,925 | 8.2 (1.0) | 5.4 (4.8) | 289 (9.9) | 134 (4.6) |
| ***LR*** | *2,678* | *8.1 (1.0)* | *4.8 (3.4)* | *158 (5.9)* | *38 (1.4)* |
| 1st grade | 1005 | 7.2 (0.5) | 5.0 (3.6) | 70 (7.0) | 18 (1.8) |
| 2nd grade | 852 | 8.2 (0.5) | 4.6 (3.2) | 41 (4.8) | 7 (0.8)_ |
| 3rd grade | 821 | 9.1 (0.5) | 4.7 (3.4) | 47 (5.7) | 13 (1.6) |
| ***ER*** | *213* | *9.0 (1.1)* | *10.9 (8.3)* | *98 (46.0)* | *64 (30.0)* |
| 1st grade | 97 | 8.3 (0.7) | 11.0 (8.0) | 98 (46.0) | 32 (33.0) |
| 2nd grade | 66 | 9.1 (0.9) | 11.6 (8.5) | 30 (45.5) | 21 (31.8) |
| 3rd grade | 50 | 10.1 (0.8) | 9.9 (8.2) | 22 (44.0) | 11 (22.0) |
| ***HR*** | *34* | *9.1 (1.1)* | *23.9 (5.8)* | *33 (97.1)* | *32 (94.1)* |
| 1st grade | 11 | 8.1 (0.7) | 23.9 (7.5) | 10 (90.9) | 9 (81.8) |
| 2nd grade | 7 | 9.1 (0.7) | 24.6 (3.4) | 7 (100.0) | 7 (100.0) |
| 3rd grade | 16 | 9.8 (1.1) | 23.6 (5.5) | 16 (100.0) | 16 (100.0) |

*SCQ = Social Communication Questionnaire; LR = Low-Risk stratum; ER = Elevated-Risk stratum; HR = High-Risk stratum*

*Diagnostic Confirmation Phase*

All screening-positive cases were scheduled to participate in the Second Phase; however, the research team encountered a low response rate (25.6%) for participation. Parents and legal representatives cited several reasons for refusal, including relocation to another city, the belief that their child does not have ASD, and the inability or unwillingness to bring a child with ASD to the assessment site. However, the most frequent reason for nonparticipation was a lack of response to phone calls and text message invitations after four contact attempts by the recruiter.

In total, 74 participants took part in the Second Phase of the study. Among them, only three were from mainstream education classes, representing the lowest response rate (6.2%) across all subgroups. In contrast, significantly higher response rates were among participants from special education and resource classes — 40.8% and 48.5%, respectively (Table 3).

**Table 3: Participant Flow and Attrition by Risk Stratum Across Study Phases**

| Stratum | Eligible | Screened, N (%) | SCQ≥11, N (%) | Attended, N (%) | ASD, N (%) |
| --- | --- | --- | --- | --- | --- |
| LR | 8,565 | 2,678 (31.3) | 289 (9.9) | 18 (6.2) | 3 (16.7) |
| ER | 292 | 175 (59.9) | *98 (46.0)* | 40 (40.8) | 27 (67.5) |
| HR | 112 | 34 (30.4) | 33 (97.1) | 16 (48.5) | 16 (48.5) |

SCQ = Social Communication Questionnaire; LR = Low-Risk stratum; ER = Elevated-Risk stratum; HR = High-Risk stratum

In total, the research team conducted 74 ADOS-2 assessments, 44 ADI-R interviews, 37 Leiter-3 and 39 Vineland-II evaluations. Based on the assessment instruments administered, ASD was diagnosed in 46 cases: three cases from LR, 27 from ER and 16 from HR).

*Prevalence*

**Table 4. Bayesian Posterior ASD Prevalence by Stratum**

|  | Posterior Mean | Median | 95% CrI |
| --- | --- | --- | --- |
| LR | 0.58 | 0.45 | 0.3-1.64 |
| ER | 21.72 | 21.6 | 17.4-27.7 |
| HR | 88.24 | 88.24 | 82.4-94.1 |
| Total | 31.4 | 30.1 | 24.0-45.8 |

*ASD = Autism Spectrum Disorder; LR = Low-Risk stratum; ER = Elevated-Risk stratum; HR = High-Risk stratum*

**Table 5. Sensitivity Analysis of ASD Prevalence under Varying Sensitivity and Specifity**

|  | **Se** | **Sp** | **Median** | **95% CrI** |
| --- | --- | --- | --- | --- |
| 1 | 0.80 | 0.65 | 2.34 | 2.01-4.00 |
| 2 | 0.85 | 0.65 | 2.28 | 2.00-3.03 |
| 3 | 0.90 | 0.65 | 2.24 | 1.98-2.95 |
| 4 | 0.95 | 0.65 | 2.18 | 1.97-2.58 |
| 5 | 0.80 | 0.70 | 2.34 | 2.04-3.87 |
| 6 | 0.85 | 0.70 | 2.31 | 2.03-3.15 |
| 7 | 0.90 | 0.70 | 2.26 | 2.02-3.17 |
| 8 | 0.95 | 0.70 | 2.21 | 2.00-2.72 |
| 9 | 0.80 | 0.75 | 2.44 | 2.09-4.36 |
| 10 | 0.85 | 0.75 | 2.32 | 2.06-2.93 |
| 11 | 0.90 | 0.75 | 2.38 | 2.06-4.35 |
| 12 | 0.95 | 0.75 | 2.29 | 2.04-4.19 |
| 13 | 0.80 | 0.80 | 2.69 | 2.15-6.32 |
| 14 | 0.85 | 0.80 | 2.72 | 2.14-7.82 |
| 15 | 0.90 | 0.80 | 2.50 | 2.11-4.94 |
| 16 | 0.95 | 0.80 | 2.33 | 2.07-4.88 |

*ASD = Autism Spectrum Disorder; Se = sensitivity, Sp = specifity*

*Sensitivity Analysis*

**Table 6. Posterior Estimates of Screening Sensitivity and Specificity from the Probabilistic Bayesian Model**

|  | **Posterior Mean** | **SD** | **Posterior CrI** |
| --- | --- | --- | --- |
| **Se** | 0.87 | 0.03 | 0.81-0.92 |
| **Sp** | 0.61 | 0.04 | 0.53-0.69 |

Se = sensitivity; Sp = specifity; SD = standard deviation

FIGURE 1

**
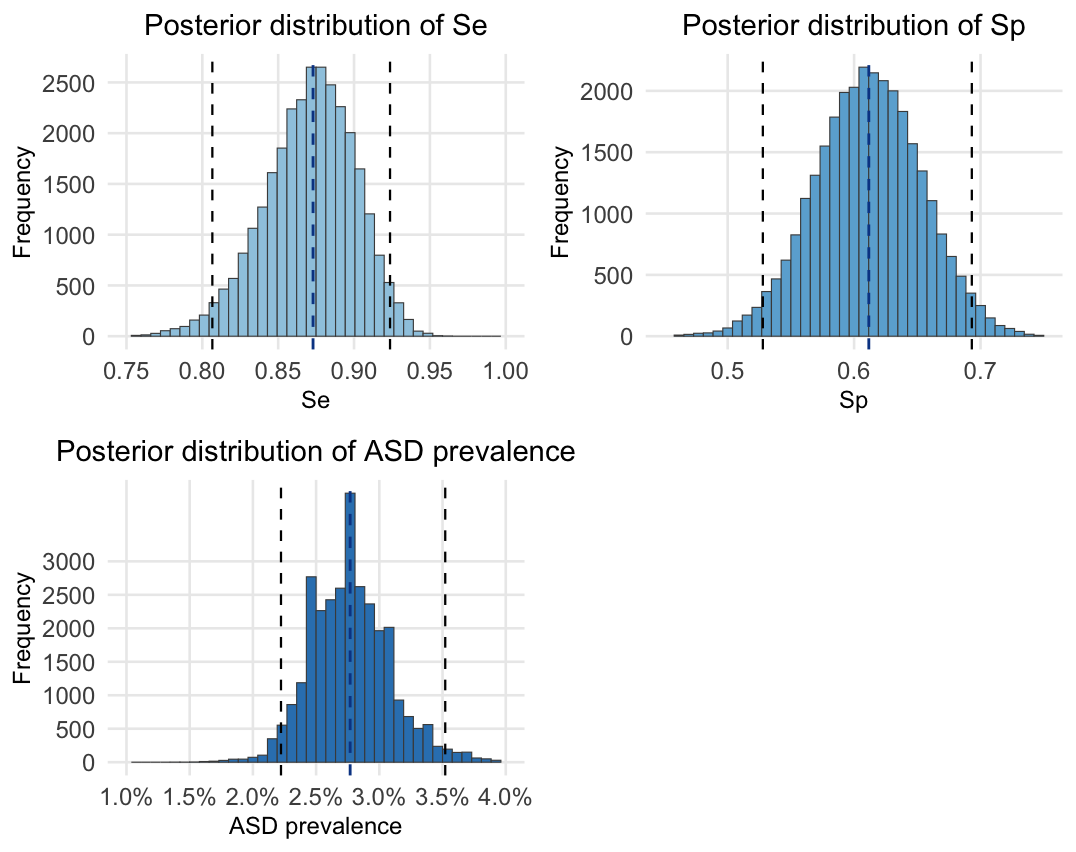
**

Posterior distribution of the post-stratified ASD prevalence under probabilistic sensitivity analysis (PSA). The Bayesian model incorporated misclassification correction with informative priors for screening sensitivity (Se ~ Beta(85,15)) and specificity (Sp ~ Beta(60,40)). The horizontal axis represents prevalence estimates, and the vertical axis shows the frequency of posterior samples across 10,000 MCMC iterations. Dashed lines indicate the posterior median (2·19%) and the bounds of the 95% credible interval (1·84%–3·05%).

*Bayesian Model Diagnostics*

For the primary regression parameters (α and β), R-hat values were 1.07, with upper 95% confidence limits of 1.12, indicating acceptable convergence across chains (Table 6).

**Table 7. Convergence diagnostics and posterior summaries for primary model parameters**

| **Parameter** | **Mean** | **SD** | **R-hat** | **R-hat_upper** |
| --- | --- | --- | --- | --- |
| alpha | -29.85 | 15.73 | 1.07 | 1.2 |
| beta | 1.70 | 0.88 | 1.07 | 1.2 |

The effective sample size (ESS) across all monitored parameters had a median of 5,753 (mean = 5,769; 1st quartile = 4,000; 3rd quartile = 8,000; maximum = 12,000). The median ESS substantially exceeded conventional minimum thresholds for stable posterior estimation (e.g., ESS > 400), supporting the numerical stability of posterior summaries.

Trace plots (Figure 2) for the primary model parameters (α and β) demonstrated adequate mixing across chains, with no visible trends, drifts, or long-term autocorrelation patterns. The chains overlapped substantially and explored stable regions of the parameter space, suggesting satisfactory convergence. Posterior density plots for both parameters were smooth and unimodal. The posterior distribution of α was left-skewed with a wide spread, consistent with its role as the intercept in the logistic model under low baseline prevalence. The posterior distribution of β was positively skewed but well-defined, indicating a stable and consistent positive association between SCQ score and latent ASD probability. Together with the Gelman–Rubin diagnostics (R-hat values close to 1.00–1.07 for primary parameters) and adequate effective sample sizes, these graphical assessments support the numerical stability and convergence of the Bayesian model.

FIGURE 2


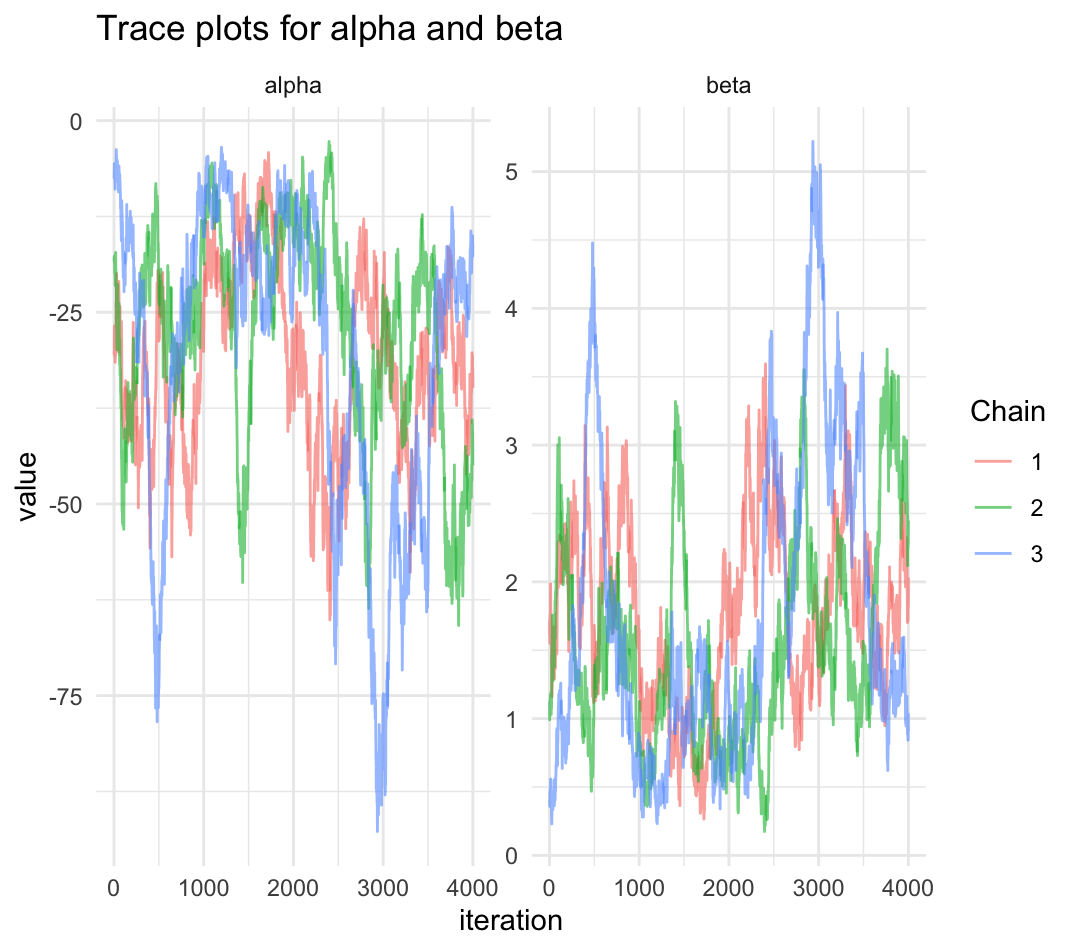


**Trace plots for regression parameters (α and β) from the Bayesian logistic misclassification model.** Markov chain Monte Carlo (MCMC) trace plots are shown for 3 chains after burn-in. The horizontal axis represents iteration number, and the vertical axis shows sampled parameter values. Visual inspection indicates adequate mixing and absence of systematic drift or chain separation, supporting convergence of the posterior simulations.

FIGURE 3


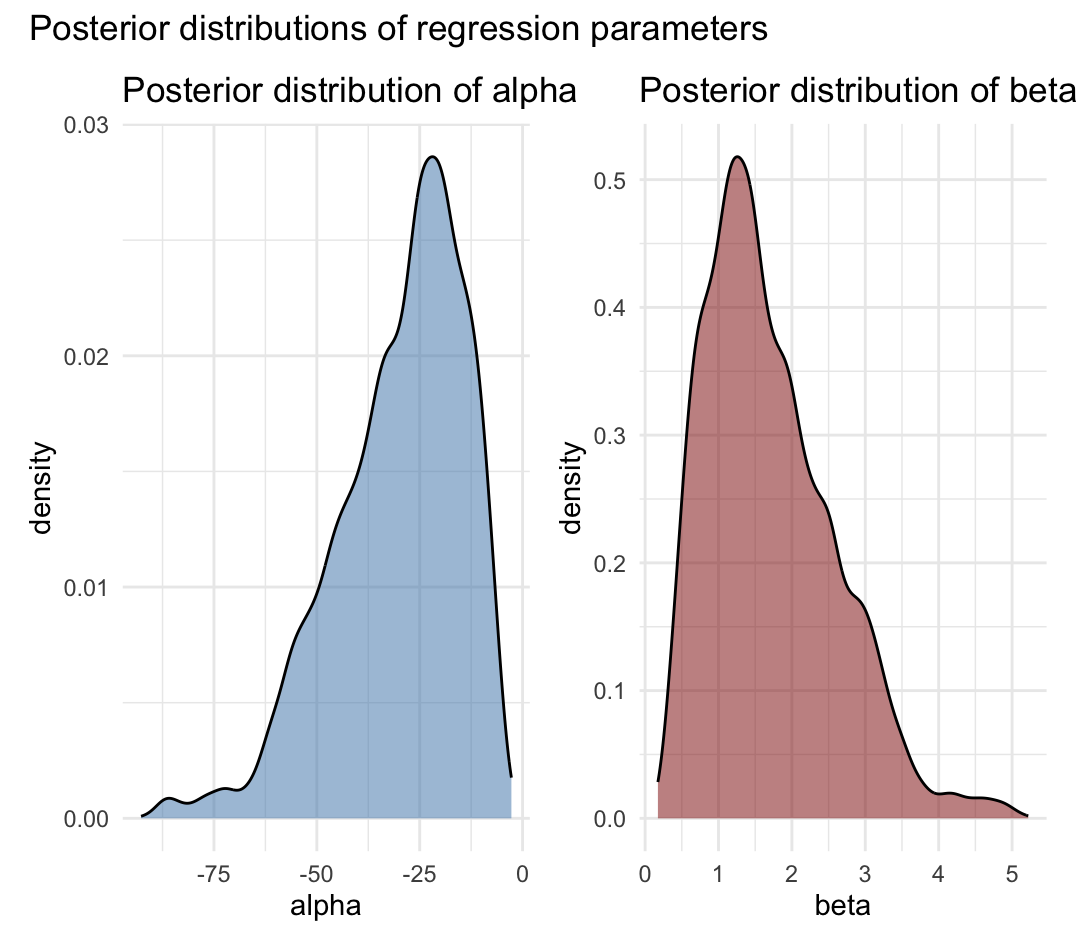


Posterior distributions of regression parameters in the Bayesian misclassification model. Kernel density plots display the posterior distributions of the intercept (α) and slope (β) parameters from the Bayesian logistic regression model used to estimate latent ASD status as a function of SCQ score. Posterior samples were obtained from 10,000 MCMC iterations following burn-in. The distributions are unimodal and approximately symmetric, supporting model stability and consistent estimation across chains.

**Alternative Calculations of ASD Prevalence, Not Included to the Final Report**

1. **Inverse Probability Weighting Based on Logistic Model of Participation (Individual-Level)**

To account for differential participation in the diagnostic assessment phase, we modeled the probability of participation using logistic regression. Two nested models were evaluated: a full model (described above) and a reduced model, which included only the SCQ result. As the full model did not improve model fit (ΔDeviance = 1.85, p = 0.40; ΔAIC < 2), the reduced model was used for subsequent calculations to estimate participation probabilities. Inverse probability weights (IPW) were calculated as the inverse of predicted participation probabilities. Weights were processed separately within each stratum to account for differences in weight distributions and case rarity. Stratified prevalence estimates were then calculated using weighted proportions of ASD cases within each stratum.

Post-stratified pooled prevalence was calculated by applying population-based weights (π) to each stratum-specific prevalence estimate:

| $P_{total}=\pi_{LR}\cdot p_{LR}+\pi_{ER}\cdot p_{ER}+\pi_{HR}\cdot p_{HR}$ | (5) |
| --- | --- |

Where *p_LR_, p_ER_, p_HR_* are stratum-specific prevalence estimates; *π_LR_, π_ER_, π_HR_* are known population proportions for each risk stratum. For the HR group, prevalence was fixed at 100% based on confirmed ASD diagnosis in all 112 children.

Ninety-five percent confidence intervals (CIs) for stratified and pooled prevalence estimates were computed using nonparametric bootstrapping with 1,000 resamples. For each stratum, the entire IPW procedure, including normalization and trimming, was repeated across bootstrap samples. Pooled CIs were obtained via propagation from the stratum-specific bounds.

*Results*

The resulting IPW-corrected ASD prevalence was 15.2 (95% CI: 0 – 36.7) per 1,000 in the LR group and 256·6 (95% CI: 169.3 – 343.3) in the ER group. For the HR group, ASD prevalence was fixed at 100% based on the inclusion criterion. The resulting pooled ASD prevalence adjusted for participation bias was 25.0 (95% CI: 8.5 – 47.6) per 1,000.

**Table 8. Comparison of logistic regression models predicting diagnostic participation**

| **Model** | **Predictors** | **AIC** | **ΔAIC** | **Deviance (χ²)** | **df** | **p-value** |
| --- | --- | --- | --- | --- | --- | --- |
| **Full** | SCQ + Age + Sex | 268.2 | 2.2 | 1.8 | 2.0 | .4 |
| **Reduced** | SCQ | 266.1 | -- | -- | -- | -- |


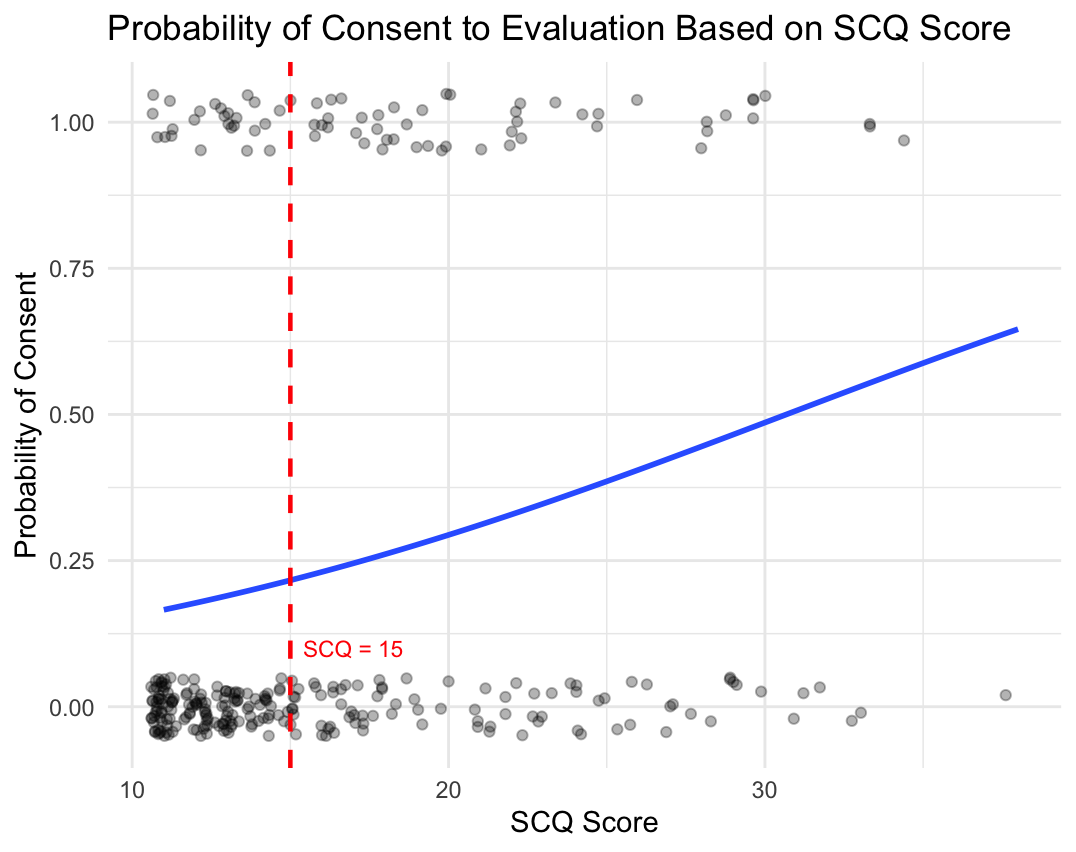


**Figure 2. Predicted probability of parental consent for clinical evaluation as a function of SCQ score, based on the reduced logistic regression model used for inverse probability weighting. Each point represents a screen-positive child from the LR or ER strata. The blue line shows the fitted logistic curve. Higher SCQ scores were significantly associated with increased probability of participation (p < 0.05).**

**Table 9. Logit-transformed and predicted probabilities of participation as a function of SCQ score**

| **SCQ Score** | **Logit_z** | **Predicted Probability** |
| --- | --- | --- |
| **11** | -1.685 | 0.156 |
| **12** | -1.589 | 0.170 |
| **13** | -1.493 | 0.183 |
| **14** | -1.397 | 0.198 |
| **15** | -1.301 | 0.214 |
| **16** | -1.205 | 0.231 |
| **17** | -1.109 | 0.248 |
| **18** | -1.013 | 0.266 |
| **19** | -0.917 | 0.286 |
| **20** | -0.821 | 0.306 |
| **21** | -0.725 | 0.326 |
| **22** | -0.629 | 0.348 |
| **23** | -0.533 | 0.370 |
| **24** | -0.437 | 0.392 |
| **25** | -0.341 | 0.416 |
| **26** | -- | -- |
| **27** | -0.149 | 0.463 |
| **28** | -0.053 | 0.487 |
| **29** | -0.043 | 0.511 |
| **30** | 0.139 | 0.535 |
| **31** | 0.235 | 0.558 |
| **32** | -- | -- |
| **33** | 0.427 | 0.605 |
| **34** | 0.523 | 0.628 |
| **35** | -- | -- |
| **36** | -- | -- |
| **37** | -- | -- |
| **38** | 0.906 | 0.712 |

SCQ = Social Communication Questionnaire

**Table 10. Logistic regression predicting participation in diagnostic evaluation**

| **Predictor** | **Estimate (β)** | **Std. Error** | **z-value** | ***p*-value** |
| --- | --- | --- | --- | --- |
| **Intercept** | -4.1 | 1.2 | -3.4 | <.001 |
| **SCQ Score** | 0.1 | 0.0 | 3.1 | .002 |
| **Age** | -0.1 | 0.3 | -0.4 | .678 |
| **Sex (Male)** | 0.2 | 0.1 | 1.3 | .186 |

SCQ = Social Communication Questionnaire; Std. – Standard

**Table 11. IPW-corrected ASD prevalence estimates**

| **Stratum** | **Population Size** | **N screen-positives** | **IPW-corrected prevalence  per 1,000** | **95% CI (per 1,000)** |
| --- | --- | --- | --- | --- |
| **LR** | 8,684 | 3 | 15.2 | 0.0 – 36.7 |
| **ER** | 292 | 27 | 256.6 | 169.3 – 343.3 |
| **HR** | 112 | 112 | 1000.0 | -- |
| **Total** | 9,088 | 142 | 25.0 | 8.5 – 47.6 |

IPW = Inverse Probability Weighting; ASD = Autism Spectrum Disorder, LR – Low Risk; ER – Elevated Risk; HR – High Risk

1. **Stratified Two-Phase Sampling Estimator with Inverse Probability Weighting (Stratum-Level)**

To compliment the analysis in the main part of the paper, we used a stratified two-phase prevalence estimation method combined with inverse probability weighting (IPW) to adjust for non-response within each stratum.

Prevalence estimates were derived by extrapolating from the sampled subgroups, incorporating both the sampling probabilities and observed participation rates. To account for estimation uncertainty, 95% confidence intervals were computed using bootstrap resampling, which integrated both sampling variability and uncertainty in diagnostic classification.

This was designed to overcome the primary constraints of the data collection, such as the exclusion of screen-negative (low SCQ) participants and the absence of formal adjustment for differential response rates at the clinical assessment stage (Table 10).

*Data Weighting and Response Bias Adjustment*

We used IPW to account for non-response bias (51.5-88.6% across strata). This method adjusts prevalence estimates by weighting participants based on the probability of completing the clinical assessment, conditional on their stratum. The estimation assumed no ASD cases in participants scoring <11 and higher likelihood of clinical assessment attendance among children with elevated SCQ scores, supported by significant between-strata differences in scores and non-response rates (Table 2).

Stratum-specific weights were defined as the **inverse of the participation probability** in clinical assessment:

(6)

$$w_{i}=\frac{n_{i}}{a_{i}}$$

Where:

$n_{i}$ is the number of children invited for clinical assessment in stratum $i$,

$a_{i}$ is the number of children who completed the assessment in stratum $i$.

To estimate the number of ASD cases in each stratum, we used:

(7)

$$\hat{D}_{i}=d_{i}\frac{n_{i}}{a_{i}}$$

Where:

$d_{i}$ is the number of ASD diagnoses observed in stratum $i$.

The total number of estimated ASD cases across strata was:

(8)

$$\hat{D}_{total}=\sum_{i=1}^{k} \left( d_{i}\frac{n_{i}}{a_{i}} \right)$$

To calculate **overall prevalence**, we divided the estimated number of cases by the total population in the study:

(9)

$$Prevalence= \frac{\hat{D}_{total}}{N}$$

Where:

*N* is the number of children in the full sampled population.

The bootstrap resampling with 1,000 iterations included resampling participants with replacement within each stratum, and recalculating the weighted number of ASD cases and prevalence per iteration.

Estimating variability in proportions using a normal approximation:

(10)

SD=$\sqrt{\frac{p\left( 1-p \right)}{n}}$

Where:

$p$ is the observed ASD prevalence in the resampled dataset,

$n$ is the effective sample size.

The bootstrap procedure incorporated sampling variability and proportion uncertainty through normal distribution resampling with observed means and calculated standard deviations. Count variability was modeled using Poisson distributions. Ninety-five percent confidence intervals were derived from the 2.5th and 97.5th percentiles of the bootstrap distribution.

**Results**

**General Population Prevalence Estimation**

To estimate the overall prevalence of ASD in the general population of the city, we aggregated the stratum-specific estimates from mainstream schools, elementary resource classes, and special education schools, each weighted by the respective population size. Summary statistics are provided in Tables 10 - 11.

The district-wide prevalence of ASD was estimated as 1.50% (95% CI: 1.38%–1.62%), corresponding to approximately 593 children diagnosed with ASD among the 39,431 children enrolled in Grades 1–3. This indicates an overall prevalence of 15.1 per 1,000 children in the target population (Table 12).

**Table 12. Summary Statistics Stratified by the School Type**

|  | **LR Population**  **(N=39,030)** | **HR Population (N=130)** | **ER Population**  **(N=271)** | **p-value** | **Total** |
| --- | --- | --- | --- | --- | --- |
| Participated in the screening | 2,676 | 34 | 213 |  | 2,923 |
| Screening score | 4.8  (±3.4) | 23.9  (±5.8) | 10.9  (±8·2) | <.001 | 5.4  (±4.8) |
| Age (yrs) | 8.1  (±1.0) | 9.1  (±1.1) | 9.0  (±1.1) | <.001 | 8.2  (±1.0) |
| **Sex** |  |  |  |  |  |
| Male | 1,381  (51.6%) | 26  (76.5%) | 140  (65.7%) | <.001 | 1,547  (52.9%) |
| Female | 1,295  (48.4%) | 8  (23.5%) | 73  (34.3%) |  | 1,376  (47.1%) |
| **Selected for assessment** | |  |  |  |  |
| Selected | 158  (5.9%) | 33  (97.1%) | 98  (46.0%) |  | 289  (9.9%) |
| Not selected | 2,518  (94.1%) | 1  (2.9%) | 115  (54.0%) | <.001 | 2,634 (90.1%) |
| **Assessment attendance** | |  |  |  |  |
| Attended | 18  (11.4%) | (48.5%) | 40  (40.8%) | <.001 | 74  (25.6%) |
| Non-responder | 140  (88.6%) | 17  (51.5%) | 58  (59.2%) |  | 215  (74.4%) |
| **ASD diagnosis** |  |  |  |  |  |
| ASD | 3  (16.7%) | 16  (100.0%) | 27  (67.5%) | <.001 | 46  (62.2%) |
| No ASD | 15  (83.3%) | 0  (0.0%) | 13  (32.5%) |  | 28  (37.8%) |

*LR = Low Risk; HR = High Risk; ER = Elevated Risk*

**Table 13*.* Summary Statistics Stratified by Attendance Status**

|  | **Non-responders** | **Attendees** | **p-value** | **Total** |
| --- | --- | --- | --- | --- |
| Number of invited | 215 | 74 |  | 289 |
| Screening score (SD) | 15.4 (±5.4) | 18.7 (±6.2) | <.001 | 16.3 (±5.8) |
| Age (yrs) | 8·4 (±1.1) | 8.8 (±1.1) | .03 | 8.5 (±1.1) |
| **Sex** |  |  |  |  |
| Male | 150 (69.8%) | 52 (70.3%) | 1.00 | 87 (30.1%) |
| Female | 65 (30.2%) | 22 (29.7%) |  | 202 (69.9%) |
| **ASD diagnosis** |  |  |  |  |
| ASD | -- | 46 (62.2%) |  | 46 (62.2%) |
| No ASD | -- | 28 (37.8%) |  | 28 (37.8%) |
| **Population** |  |  |  |  |
| LR | 140 (65.1%) | 18 (24.3%) | <.001 | 158 (54.7%) |
| HR | 58 (27.0%) | 40 (54.1%) |  | 98 (33.9%) |
| ER | 17 (7.9%) | 16 (21.6%) |  | 33 (11.4%) |

*LR = Low Risk; HR = High Risk; ER = Elevated Risk; SD = Standard Deviation*

**Table 14. Prevalence of ASD: overall and in different educational settings and overall**

| **Population** | **Population Size** | **Point Prevalence** | **Count of cases in the district** | **Rate per**  **1,000** | **NT-to-ASD ratio** |
| --- | --- | --- | --- | --- | --- |
| **LR** | 39,030 | 1.0%  (95%CI 0.9 – 1.1) | 382,49 | 9.8 | 101.0:1 |
| **HR** | 130 | 97.1%  (95%CI 94.2 – 100.0) | 126,23 | 970.6 | 0.0:1 |
| **ER** | 271 | 31.1%  (95%CI 25.4 – 36.2) | 84,01 | 310.6 | 2.2:1 |
| **General population** | 39,431 | 1·5%  (95%CI 1.4 – 1.6) | 592,73 | 15.1 | 10.2:1 |

*LR = Low Risk; HR = High Risk; ER = Elevated Risk; NT = Neuro-Typical; ASD – Autism Spectrum Disorder*

**References**

1. Lwanga SK, Lemeshow S, World Health Organization. Sample Size Determination in Health Studies: A Practical anual. Geneva: World Health Organization; 1991.
2. Naing L, Winn T, Rusli BN. Practical issues in calculating the sample size for prevalence studies. Arch Orofac Sci. (2006) 1:9-14.
3. Pourhoseingholi MA, Vahedi M, Rahimzadeh M. Sample size calculation in medical studies. Gastroenterol Hepatol Bed Bench. (2013) 6(1):14-17.
4. Rutter M, Bailey A, Lord C. The Social Communication Questionnaire: Manual [Social'no-kommunikativnyj oprosnik. Rukovodstvo]. Moscow: Guinty Psychometrics Rus. (2013).
5. Allen CW, Silove N, Williams K, Hutchins P. Validity of the Social Communication Questionnaire in assessing risk of autism in preschool children with developmental problems. J Autism Dev Disord. (2007) 37(7):1272-1278. doi:10.1007/s10803-006-0272-1
6. Lord C, Rutter M, DiLavore P, Risi S, Gotham K, Bishop S. *ADOS-2 Autism Diagnostic Observation Schedule–2nd Edition: Manual* [Plan diagnosticheskogo obsledovaniya pri autizme. Rukovodstvo]. Moscow: Guinty Psychometrics Rus. (2013).
7. Rutter M. Autism Diagnostic Interview-Revised (ADI-R): Manual [Interv'yu pri diagnostike autizma. Rukovodstvo]. Moscow: Guinty Psychometrics Rus. (2016).
8. Roid GH, Miller LJ, Koch C. Leiter International Performance Scale, Third Edition: Manual [Lejter-3 – Mezhdunarodnye shkaly produktivnosti. Rukovodstvo]. Moscow: Guinty Psychometrics Rus. (2014).
9. Ovchinnikova IV, Zhukova MA, Grigorenko EL. Approbation of Vineland Adaptive Behavior Scales (VABS) on a Russian-speaking sample [Aprobacija metodiki Vineland Adaptive Behavior Scales (VABS) na russkojazychnoj vyborke]. Voprosy Psychologii. (2018) 6:134-146.
10. Gelman A, Rubin DB. Inference from iterative simulation using multiple sequences. Stat Sci. 1992; 7(4):457–472. doi:10.1214/ss/1177011136
11. Vehtari A, Gelman A, Simpson D, Carpenter B, Bürkner PC. Rank-normalization, folding, and localization: An improved R̂ for assessing convergence of MCMC. Bayesian Anal. 2021;16(2):667–718. doi:10.1214/20-BA1221
12. Gelman A, Carlin JB, Stern HS, Dunson DB, Vehtari A, Rubin DB. Bayesian Data Analysis. 3rd ed. Boca Raton (FL): CRC Press; 2013.and predicted proportions, following standard Bayesian model-checking procedures. doi:10.1201/b16018
13. R Core Team and contributors worldwide. R Base Packages: base, compiler, datasets, grDevices, graphics, grid, methods, parallel, splines, stats, stats4, tcltk, tools, and utils. Vienna: R Foundation for Statistical Computing. (2023).
14. Wickham H. plyr: Tools for Splitting, Applying and Combining Data [computer program]. Version 1.8.9. 2008. [Accessed June 26, 2025] <https://doi.org/10.32614/CRAN.package.plyr>
15. Canty A, Ripley B. Boot: Bootstrap R (S-Plus) Functions [computer program]. Version 1.3-28.1. 2022. [Accessed June 26, 2025.] <https://cran.r-project.org/web/packages/boot/boot.pdf>
16. Plummer M. rjags: Bayesian Graphical Models using MCMC [computer program]. 2008. Accessed June 26, 2025. <https://doi.org/10.32614/CRAN.package.rjags>
17. Plummer M, Best N, Cowles K, Vines K. CODA: convergence diagnosis and output analysis for MCMC. R News. (2006) 6:7-11.
18. Wickham H. ggplot2: Elegant Graphics for Data Analysis. New York: Springer-Verlag. (2016).
